# Medical applications of RNA interference (RNAi)

**DOI:** 10.1186/1753-6561-7-S2-K21

**Published:** 2013-04-04

**Authors:** Tiago Campos Pereira, Iscia Lopes-Cendes

**Affiliations:** 1 Department of Biology, Faculty of Philosophy, Sciences and Languages of Ribeirao Preto, University of Sao Paulo - USP, Ribeirao Preto, SP, Brazil; 2 Department of Medical Genetics, Faculty of Medical Sciences, University of Campinas - UNICAMP, Campinas, SP, Brazil

## 

During its long and rich history, medical sciences have undergone several changes of paradigms. In this quest to develop more potent drugs, less invasive surgical techniques and more sophisticated diagnostic approaches (among other goals) medicine has contributed decisively to our well being and longevity. Some of these major accomplishments, which altered the course of humanity are: the development of antibiotics, organ transplantation and radiography. Medicine has recently reached a new hallmark: RNA interference (RNAi), a Nobel prize winning technology which promises to promote medical care at the molecular level, by regulating genes in our favor.

RNAi was first successfully used in *C. elegans*, a laboratory model species, by microinjecting dsRNAs in the worm’s gonad. However, the works that laid the foundations for the development of the technique were initially done in plants and fungi. RNAi was accidentally discovered, when white transgenic petunias were obtained instead of deep purple ones [1]; a similar paradoxal result was obtained when white transgenic colonies of *Neurospora sp* were observed in place of orange ones [2]. These findings revealed the existence of an unknown potent mechanism of gene regulation, which could be tamed for diverse uses, from basic science, agriculture, animal health and medicine.

From these seminal observations, during a period of nearly 10 years, researchers aimed at characterizing, at the genetic and biochemical levels, the process of RNAi. By the year 2001, scientists were ready to perform the initial analyses in human cells [3]. Since then, RNAi has been used as a tool in molecular biology, allowing potent and specific gene silencing. This is achieved by the introduction of double-stranded RNA molecules (dsRNAs), which ultimately lead to the degradation of messenger RNAs (mRNAs), *i.e.*, gene silencing. The molecular mechanism behind RNAi requires the activation of an endogenous pathway which is present in almost all eukaryotic cells. This pathway is responsible for detecting long dsRNAs and, subsequently, promoting their cleavage by an enzyme called dicer, into small duplexes of approximately 21-25 bp called siRNAs (small interfering RNAs). Alternatively, siRNAs can be directly introduced into the cytoplasm of eukaryotic cells. This second approach is required in mammalian cells, since dsRNAs longer than 30 bp induce apoptosis. siRNAs are subsequently transferred to a complex called RISC (RNA-induced silencing complex), where one strand of the duplex is destroyed and the other remains intact (designated guide strand). RISC then searches the cytoplasm for mRNA molecules with sequence complementarity to the guide strand. Once a mRNA with full complementarity is found, slicer (an endoribonuclease which is part of RISC) performs the cleavage of target mRNA [4].

Therefore, RNAi can be used as a technique that takes advantage of an endogenous biochemical pathway, directing it to the destruction of specific target transcripts (gene silencing). In addition, RNAi can be used to produce transgenic animals (known as knockdown animals), resembling the null phenotype (^-^ / ^-^). Additionally, since RNAi may be tamed to promote silencing efficiencies ranging from 0.1% to 100% [5,6], animals with partial phenotypes , known as hypomorfic, may generated. These hypomorfic individuals are very informative since they reveal intermediary and unprecedented phenotypes and because the null genetic construction may happen to be unviable [7].

Currently, most studies use RNAi as a tool for reverse genetics (identification of gene function), but the applications are numerous: **i)** disease control (viruses [8]; bacterial diseases [9]; parasites, [10]); genetic [11]; tumors [12], **ii)** production of animals of commercial interest [13] and **iii)** production of animal models for research use [14]. Other possible future applications include: control of drug consumption [15], pain relief [16], modulation of sleep [17], among many others.

Another relevant feature of RNAi is its temporary effect - long lasting knockdown is only observed in *C. elegans* and a few other species. Therefore, in order to study genes with constitutive expression, it is necessary to combine long-term strategies such as the production of transgenic animals encoding shRNAs (similar to siRNAs) or the use of viral vectors. On the other hand, this transitory characteristic can also be used as an advantage, when one considers RNAi as a therapetical strategy.

Furthermore, the control of gene expression via RNAi can be modulated in time and directed to specific cells or organs. The identification of viral proteins capable of suppressing RNAi, such as p19 [18] allows for future applications, as well as the use of inducible promoters (high temperature, drugs).

All these new developments have recently led to the first published human clinical trials with very promising results [19].

## Competing interests

There are no competing interests in this presentation.
